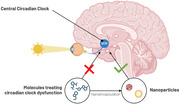# Improving the Clinical Translation of Circadian Clock Dysfunction Treatments in Alzheimer’s Disease with Nanoformulations

**DOI:** 10.1002/alz.095465

**Published:** 2025-01-09

**Authors:** Marion Le Meur, Paolo Blasi, Valle Palomo Ruiz

**Affiliations:** ^1^ IMDEA Nanociencia, Madrid Spain; ^2^ Università di Bologna, Bologna Italy; ^3^ Centro de Investigaciones Biomédicas en Red en Enfermedades Neurodegenerativas (CIBERNED), Madrid Spain; ^4^ Unidad de Nanobiotecnología Asociada al Centro Nacional de Biotecnología (CNB‐CSIC), Madrid Spain

## Abstract

**Background:**

About half of the patients suffering from Alzheimer’s disease (AD) display sleeping disorders. Disruptions in the central circadian clock (CC), located in the brain, accelerate AD pathogenesis, making the CC a promising target. In preclinical trials, this strategy have shown efficacy but clinical results are inconsistent. The presence of the blood‐brain‐barrier (BBB) and the poor pharmacokinetic profile of candidate drugs are an explanation of their poor clinical translation. Their nanoencapsulation is a potential solution to enhance brain penetration. Nanoparticles (NP) can further be formulated in pulsatile drug delivery systems (PDDS), delivering the drug at a specific time of the day to restore the circadian rhythmicity.

**Method:**

Melatonin was encapsulated in stealth polymeric NP of chitosan and PLGA, proteic NP of BSA and nanostructured lipid carriers (NLC), functionalized with brain penetrating retroenantiomer peptides, angiopep‐2 and transferrin, and labelled by a fluorophore. The NP were characterized by dynamic light scattering (DLS), zetametry, encapsulation efficiency and release profile. The best NP were selected based on their potential for passive brain permeation, using a parallel artificial membrane permeability assay (PAMPA) with brain polar lipid extract, and tracked by DLS and fluorescence. Their neuroprotective effect and toxicity were evaluated on SH‐SY5Y cell line.

**Result:**

A repeatable library of NP was obtained with a size ranging from 50 to 150 nm and a zeta potential from ‐30 to 10 mV. No toxicity was observed at concentration above clinical brain concentrations. The PAMPA highlighted a better crossing ability of some NP over other types.

**Conclusion:**

This study allowed us to rationally select different types of nanoparticles over others which will be further optimized using industrial methods. A validation of the active transport triggering will be made on a BBB‐on‐a‐chip model. After validation, the NP effect on CC rhythmicity will be evaluated on organotypic brain culture and be formulated in dissolving microneedles.